# SCHIZOPHRENIC DREAMS: A Brief Description of Sleep Disturbances in Schizophrenia

**DOI:** 10.1192/j.eurpsy.2024.1611

**Published:** 2024-08-27

**Authors:** S. Jesus Magueta, A. L. Costa, G. Simões, A. I. Gomes, C. Madaíl Grego, P. Garrido

**Affiliations:** Departamento de Psiquiatria e Saúde Mental, Centro Hospitalar do Baixo Vouga, Aveiro, Portugal

## Abstract

**Introduction:**

Shakespeare wrote that “We are such stuff as dreams are made on; and our little life is rounded with a sleep.” Sleep is a fundamental part of our being, so much so, humans tend to spend one third of their lives in this immobile and vulnerable state. Disorders of sleep have been the target of much scientific curiosity and investigation, with inumerous articles, reports and books dedicated to the theme. The bidirectional relationship between psychiatric disorders and those of sleep is also well described. Schizophrenia is a heterogenous psychiatric disorder which is often associated with sleep disturbances of various kinds.

**Objectives:**

The authors aim to briefly explore the relationship between schizophrenia and sleep disturbances. Potential underlying mechanisms and risk factors, as well as therapeutic interventions will be addressed.

**Methods:**

The authors conducted a brief non-structured narrative literature review using articles published in the Medline/Pubmed, ScienceDirect and Google Scholar databases. The keywords used during the research, alone or in combination, included: sleep disturbance, sleep disorder and schizophrenia.The studies consulted in this work included: cross-sectional studies, cohort studies, literature reviews and clinical case reports. Works that were included, were written in the English language and deemed as pertinent to the explored theme.

**Results:**

Although sleep disturbances do not make up part of the criteria formal diagnosis of schizophrenia, they are present in approximately 80% of those with the condition and have been identified as a common symptom in prodromic clinical pictures. The problems in sleep are as heterogenous as the presentations in schizophrenia, ranging from insomnia, restless legs syndrome, obstructuve sleep apnea, circadian rhythm disfunctions to hypersomnia. Sleep has been identified as fundamental for the reparation and restoration of various bodily systems, it is no surprise that sleep irregularities, especially in schizophrenia, can significantly reduce quality of life and promote deterioration. Some studies have stated the role that D2 receptors have in the classic symptoms of schizophrenia as well as on sleep disturbances. Second-generations antipsychotics have not only demonstrated much promise on psychotic symptoms, but they appear to aid in sleep regulation and quality.

**Conclusions:**

Sleep is fundamental for mental health. Various sleep disturbances have been identified in those suffering with schizophrenia. Slepe disturbances have been associated with worse outcomes, more florid clinical pictures and significant deterioration. Thus, bettering sleep quality in these patients, would permit better health outcomes which are fundamental in those who live with schizophrenia.

**Disclosure of Interest:**

None Declared

